# Virtual screening, optimization and molecular dynamics analyses highlighting a pyrrolo[1,2-a]quinazoline derivative as a potential inhibitor of DNA gyrase B of *Mycobacterium tuberculosis*

**DOI:** 10.1038/s41598-022-08359-x

**Published:** 2022-03-18

**Authors:** Juan Marcelo Carpio Arévalo, Juliana Carolina Amorim

**Affiliations:** Academic Unit of Health and Wellness, Catholic University of Cuenca, Cuenca, Azuay Ecuador

**Keywords:** Computational biology and bioinformatics, Drug discovery, Microbiology, Structural biology, Infectious diseases

## Abstract

Tuberculosis is a disease that remains a significant threat to public health worldwide, and this is mainly due to the selection of strains increasingly resistant to *Mycobacterium tuberculosis*, its causative agent. One of the validated targets for the development of new antibiotics is DNA gyrase. This enzyme is a type II topoisomerase responsible for regulating DNA topology and, as it is essential in bacteria. Thus, to contribute to the search for new molecules with potential to act as competitive inhibitors at the active site of *M. tuberculosis* DNA gyrase B, the present work explored a dataset of 20,098 natural products that were filtered using the FAF-Drugs4 server to obtain a total of 5462 structures that were subsequently used in virtual screenings. The consensus score analysis between LeDock and Auto-Dock Vina software showed that ZINC000040309506 (pyrrolo[1,2-a]quinazoline derivative) exhibit the best binding energy with the enzyme. In addition, its subsequent optimization generated the derivative described as PQPNN, which show better binding energy in docking analysis, more stability in molecular dynamics simulations and improved pharmacokinetic and toxicological profiles, compared to the parent compound. Taken together, the pyrrolo[1,2-a]quinazoline derivative described for the first time in the present work shows promising potential to inhibit DNA gyrase B of *M. tuberculosis*.

## Introduction

*Mycobacterium tuberculosis* is the infectious agent that causes the disease called tuberculosis (TB) that primarily affects the lungs, but is not necessarily limited to this organ^1^. Although is treatable, estimates by the World Health Organization indicate that adequate annual resources for prevention, diagnosis, and treatment of TB would be US$ 13 billion annually until 2022, however it is still far from being reached. The countries with the largest increase in the number of people diagnosed with TB between the years 2019 and 2020 include India (41%), Indonesia (14%), and the Philippines (12%)^2^. These countries contributed to alarming number of approximately 10 million people manifesting the disease worldwide (similar numbers to the year 2012), of which approximately 1.3 million died from direct causes of TB in 2020^2^. The main causes of the alarming number of deaths are inadequate diagnostic methods, limited universal health coverage, and the emergence of strains resistant to the main antibiotics used. The so-called multidrug-resistant strain of TB (MDR-TB) is defined as a strain resistant to the two first-line drugs in the treatment of the disease (e.g. rifampicin and isoniazid). On the other hand, the extensively drug-resistant strain of TB (XDR-TB) is a type of DR-TB strain that is resistant to first-line drugs and additionally resistant to fluoroquinolones and one of the second-line injectable drugs (e.g. kanamycin, amikacin, and capreomycin). Last, the extremely-drug-resistant strain of TB (XXDR-TB), is resistant to all previously described antibiotics^1,3,4^.

The occurrence of strains resistant to existing antibiotics increases the need to search for new treatment options for infectious diseases^5^. Among the main paths to be explored are the development of new synthetic or semi-synthetic drugs, structural modifications of existing drugs, and also the isolation of natural products (NPs), generally followed for optimizations of their chemical structures. These NPs can be from groups of living species ranging from microorganisms, animals, to plants, which have both terrestrial and marine origins^6^. The structural complexity of NPs is one of the most relevant features of this group of molecules, which allows for the exploration of a multitude of different scaffolds^7,8^, which has led to the discovery of several of the most successful antibiotics available to date^9^.

Bacterial topoisomerases belong to a structurally and mechanistically diverse group of enzymes responsible for regulating DNA topology^10^. According to the catalysis mechanism of breaking and reconnection of DNA, these enzymes are classified into two types: type (I) those responsible for reactions involving single-strand breaks and type (II) those responsible for double-strand breaks in DNA^10^. Among the pharmacological attractive and validated targets for antibiotic development are DNA gyrases^11–13^, as demonstrated by numerous in silico^14–16^ and in vitro studies^17–19^. This type II topoisomerase is a heterotetramer composed of two subunits, GyrA and GyrB, which when active form an A2B2 complex. This is the only enzyme that can catalyze in an ATP-dependent manner the introduction of negative supercoils into the DNA of *M. tuberculosis*^11^.

To expand the existing knowledge about the ability of NPs to act as competitive inhibitors against the ATPase domain of GyrB of *M. tuberculosis* (*Mt*GyrB), the present work started with a database of 20,098 NPs structures retrieved from the UCSF ZINC15 database. After applying filters that met the Lipinski rule of five and remove Pan Assay Interference Compounds (PAINS) and covalent inhibitors, molecular docking-based virtual screenings (MDVS) were conducted with 5462 ligands using LeDock, PLANTS, and Auto-Dock Vina (hereafter called Vina) software to select the most promising compound. Subsequently, the chemical structure of this ligand was optimized and its pharmacokinetic and toxicological properties were evaluated. In addition, molecular dynamics (MD) analyses were performed to further study the interactions between the ligand and the enzyme, as well as the stability of this complex. The results generated in the virtual screening revealed ZINC4030309506 ((3Ar)-N-(3-acetamidophenyl)-1,5-dioxo-3,4-dihydro-2H-pyrrolo[1,2-a]quinazoline-3a-carboxamide), a pyrrolo[1,2-a]quinazoline derivative as the molecule that exhibited the highest binding energy with *Mt*GyrB. After two structural optimization steps, the new derivative showed higher binding energy and improved stability with *Mt*GyrB, as well as a better pharmacokinetic and toxicological profile than ZINC403030309506. Overall, the results of the present in silico study reveal a new synthetically accessible pyrrolo[1,2-a]quinazoline derivative with the potential to inhibit *Mt*GyrB and merit further studies aimed at evaluating its antibacterial effect.

## Results and discussion

### Crystal selection, modeling of 3ZKB, and redocking

For the present work, the 3ZKB GyrB crystal was selected over the two other crystals of *M. tuberculosis* available on the RCSB Protein Data Bank: 3ZKD and 3ZM7. Although these two crystals also possess the N-terminal ATPase domain of *Mt*GyrB they have inferior stereochemical quality^20^ compared to the selected crystal, a critical feature for in silico experiments. In addition, the region between amino acids 216–239 (distant from the active site of the protein, Fig. S1A highlighted in red) corresponding to a 3ZKB loop was rebuilt due to its low electronic density. The analysis of the best model, among the 500 built using the Modeller tool (hereafter called 3ZKBL), showed a zDOPE score of − 0.8 and a root mean square deviation (RMSD) of − 1.948. The Ramachandran plot obtained with the MolProbity tool showed that the selected model has 12 residual outliers (Fig. S1B), better results than the original crystal, which had 34 outliers. The result of the ERRAT tool shows that the overall quality factor of the model is 85.67, the PROVE tool indicates that the selected model presents 3.4% of buried atoms for this protein, while VERIFY shows that 87.92% of amino acid residues have a 3D–1D score ≥ 0.2. In overall, these analyses show very similar values in quality compared to the original crystal.

Molecular docking is among the most widely used methods in drug discovery, which has driven the development of several docking algorithms and scoring functions^21^. However, their performances can vary greatly depending on the ligand–protein complex under study. For this reason, the first step of the present work was to redock the co-crystallized ligand of 3ZKB, ANP, to evaluate the performance of LeDock, PLANTS, and Vina to reproduce its crystallographic pose. The redocking using LeDock showed an RMSD value of 0.6 Å, while PLANTS and Vina showed RMSD values of 1.8 and 1.0 Å, respectively (Fig. S1C-E). These results show that LeDock has a better ability to reproduce the ANP pose in the 3ZKBL ATPase domain. However, although the RMSD values of PLANTS and Vina were higher, they also have performances that are considered acceptable (RMSD < 2.0 Å).

### Validation of the molecular docking protocol to virtual screening

To obtain higher hit rates in virtual screening, the consensus approach is widely accepted, which involves screening the same data set with more than one software and then selecting the best ranked molecule in consensus among the software used^22,23^. Fig. S2A-C and Table S1 present comparatively the scoring profiles of the three virtual screenings performed with the 5462 ligands and with the four controls: ANP, ATP, and two inactive compounds tested by the *E. coli* GyrB ATPase inhibition assay (2-chloro-5-nitroaniline and 4-amino-1H-imidazole-5-carboxamide). The Pearson correlation analysis between LeDock and Vina scores was 0.57 (*p* < 0.001), between LeDock and PLANTS scores was 0.29 (*p* < 0.001), while the scores between PLANTS and Vina showed a correlation of 0.28 (*p* < 0.001), Fig. S2D.

Because of its higher correlation, to select the most promising ligand the rank-by-rank approach^23^ was used between LeDock and Vina scores. For this, the 5462 ligands were numbered according to their binding energy scores, and their positions were used to select the best-ranked ligand by both software (Table S2). Finally, to define the software to perform the remaining analyses, LeDock and Vina were used to conduct additional virtual screening of *Mt*GyrB inhibitors with known pKi, and these values were correlated with the docking scores of the respective software. For this, 140 inhibitors with pKi values obtained from in vitro* Mt*GyrB inhibition assays were retrieved from the UCSF ZINC15 database and submitted to the virtual screenings with the same parameters previously used. These results show that the best correlation between in silico and in vitro data was obtained with LeDock (0.52; *p* < 0.05) compared to the performance with Vina (0.18; *p* < 0.05), Fig. S3 A-C. Collectively, these results, as well as the better performance of LeDock for redocking of ANP were the basis for the choice of this software for subsequent docking analyses, and for the selection of the ligand pose used in MD analyses.

**Figure 1:**
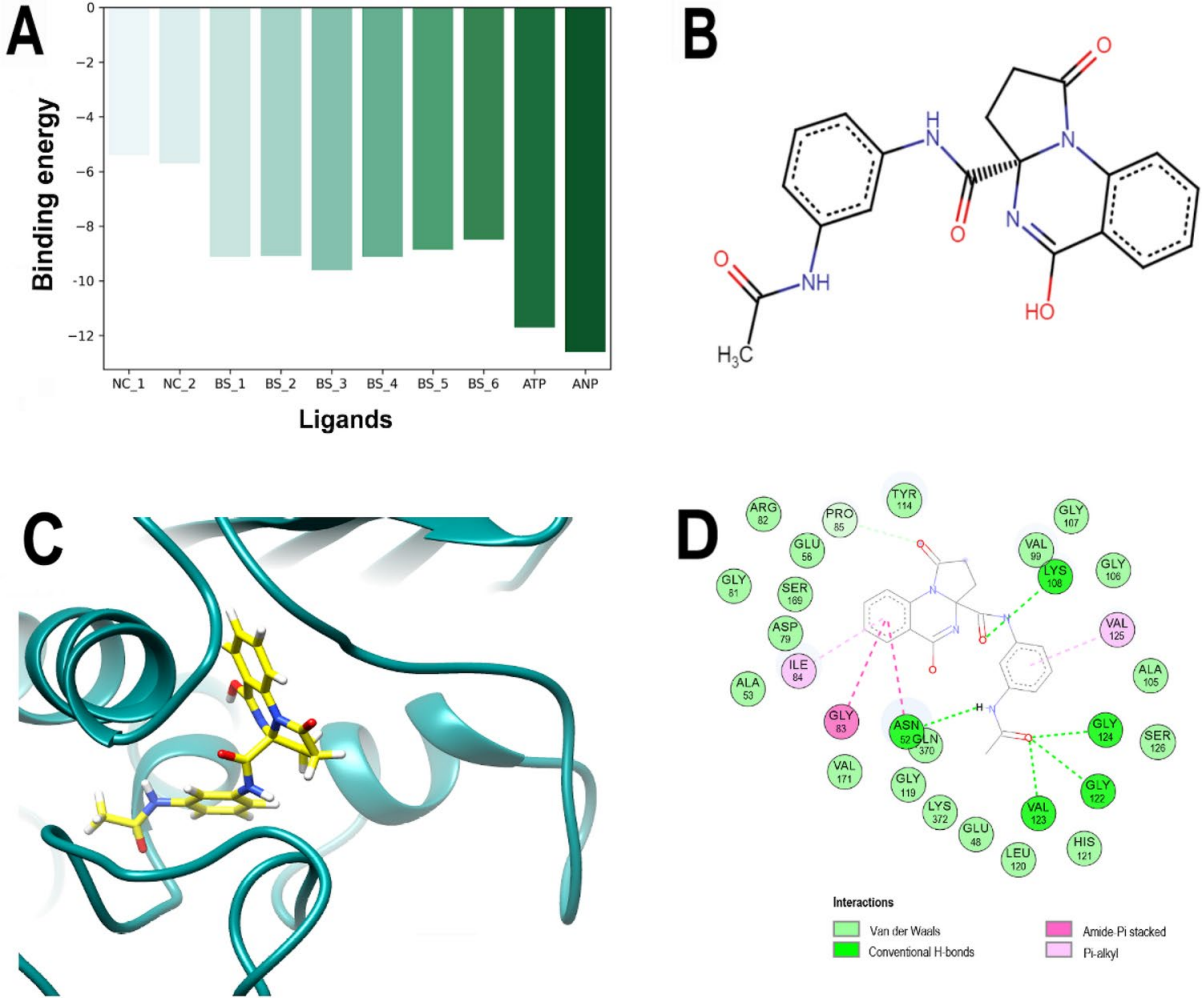
Best scored ligands, chemical structure, and analysis of the best-predicted docking pose of PQd calculated by LeDock. (**A**) Comparison of the binding energies among best scored natural products BS-1 (ZINC000040309506), BS-2 (ZINC000001529323), BS-3 (ZINC000012462127), BS-4 (ZINC000008577218), BS-5 (ZINC000064799791) and, BS-6 (ZINC000065074826), as well as ANP (ZINC000008660410), ATP (ZINC000011524400), and two negative controls NC-1(ZINC000001688375, 2-chloro-5-nitroaniline) and NC-2 (ZINC000003861263, 4-amino-1H-imidazole-5-carboxamide) calculated by LeDock. The ZINC code was omitted from the figure legend only for simplification. (**B**) Chemical structure of PQd. (**C**) 3D representation of the 3ZKBL-PQd complex. (**D**) 2D interaction diagram of the 3ZKBL-PQd complex.

Therefore, considering the LeDock results, the best scored (BS) molecules from this dataset were: ZINC000040309506 (hereinafter called PQd), ZINC000001529323, ZINC000012462127, ZINC000008577218, ZINC000064799791, and ZINC000065074826, also compared to controls, Fig. 1A. In particular, PQd, a pyrrolo[1,2-a]quinazoline derivative (Fig. 1B) was ranked sixth by Vina (− 11.5 kcal/mol) and 26th by LeDock (− 9.12 kcal/mol) among the 5462 ligands and was therefore selected for further analyses. Remarkably, although there are no previous studies on the antibacterial potential of this molecule, some pyrrolo[1,2-a]quinazoline derivatives have shown activity against bacteria such as *Micrococcus luteus*, *Pseudomonas aeruginosa,* and *Bacillus subtilis*^24^, even though its mechanism of action has not been elucidated.

### Molecular docking analyses

By analyzing the pose with the best binding energy of PQd calculated by LeDock, it is possible to observe that its acetamidophenyl moiety is buried in the active site of 3ZKBL, while its pyrrolo[1,2-a]quinazoline scaffold is almost perpendicular and oriented towards the outside of the catalytic site (Fig. 1C). Regarding interactions, Fig. 1D shows that PQd establishes one hydrogen bond (H-bond) with LYS108 through the oxygen atom of its carboxamide linker, while the acetamidophenyl moiety participates in H-bonds through its NH with ASN52 and the carbonyl group with the NH of the peptide bonds of GLY122, VAL123, and GLY124. The non-polar interactions involve the aromatic ring with VAL125, as well as the quinazolinic benzene ring with ILE84 (π–alkyl), which also form two interactions with ASN52 and GLY83 (amide-π staking)^25,26^.

### Structural optimization of PQd

**Figure 2:**
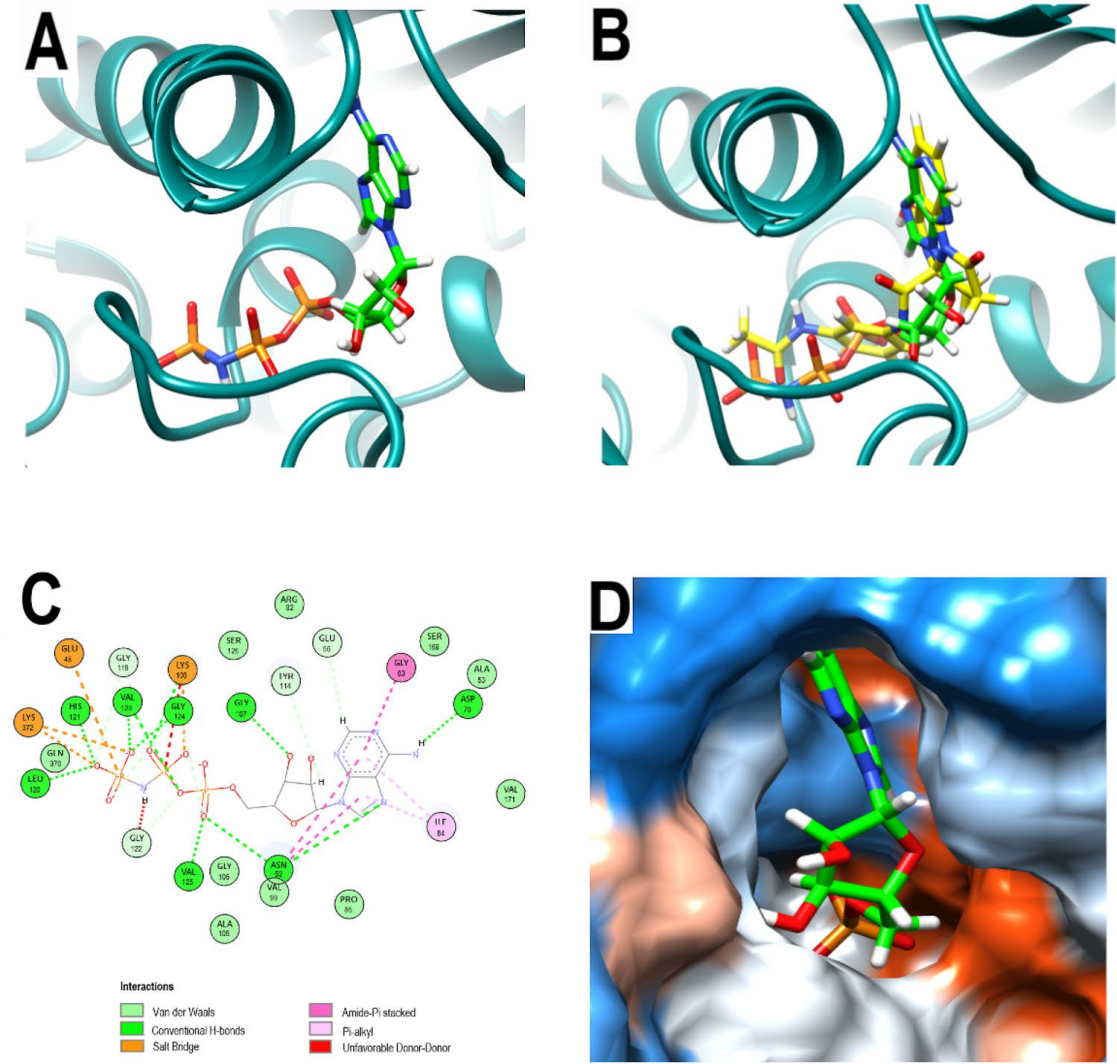
Analysis of the crystallographic pose of ANP. (**A**) 3D representation of the 3ZKB-ANP complex. (**B**) 3D representation of 3ZKBL-ANP-complex superimposed with PQd (yellow backbone). (**C**) 2D interaction diagram of 3ZKBL-ANP complex. (**D**) Hydrophobic surface representation of 3ZKBL complexed with ANP*.*

To optimize the structure of PQd, the first step was to obtain information from the crystallographic pose of ANP, a non-hydrolysable ATP analogous, in 3ZKB (Fig. 2A,D). Notably, as shown the Fig. 2B, the orientation of PQd in the catalytic site of 3ZKBL closely resembles that of ANP. The interaction pattern of the 3ZKBL-ANP complex depicted in Fig. 2C reveals several non-polar interactions of the purine backbone with ILE84 (π-alkyl), with ASN52, and with GLY83 (amide-π staking). Furthermore, ANP establishes H-bonds through the imidazolic nitrogen (with ASN52), the amine group (with ASP79), and the 3´-hydroxyl of the ribose (with the peptide bond of GLY107). However, most of the H-bonds involve the alpha phosphate (with ASN52 and with the peptide bond of VAL125), the oxygen atom of the anhydride bond (with the peptide bond of VAL123), the beta phosphate (with LYS108), and the gamma phosphate (with LEU120 and with the peptide bond of HYS121, VAL123, and GLY124). Furthermore, beta and gamma phosphates also form electrostatic interactions involving GLU48, LYS108, and LYS372. Taken together, these results show that, in addition to having a similar orientation in the active site of 3ZKBL, PQd and ANP also establish some similar interactions. However, it is evident that phosphate groups in the ANP structure favor the formation of electrostatic interactions and increase the number of H-bonds.

Based on the above analyses, PQd derivatives were generated by retaining its carbonyl group of the acetamidophenyl moiety, which establishes several H-bonds, but substituting the amino and the methyl groups with phosphate to evaluate their impact on binding energy (Table 1). In this regard, the modification of chemical structures with phosphate groups is a strategy that has allowed the development of multiple drugs including some antibiotics^27^. The results show that the derivative obtained from the substitution of methyl by phosphate group (hereafter called PQNCP) underwent a detrimental effect on the binding energy. On the other hand, the substitution of -NH by a phosphate group generates a PQd derivative (hereafter called PQP) with slightly better binding energy compared to the parent compound.

**Table 1:** Chemical structures and binding energies of PQd and its derivatives.

Structure	Binding energy kcal/mol
	**− 9.12**
	**− 7.90**
	**− 9.25**

The binding energies were calculated with LeDock. The names of the compounds, PQNCP and PQP were assigned arbitrarily based on the position of the phosphate group substituted in the acetamidophenyl moiety.

**Figure 3:**
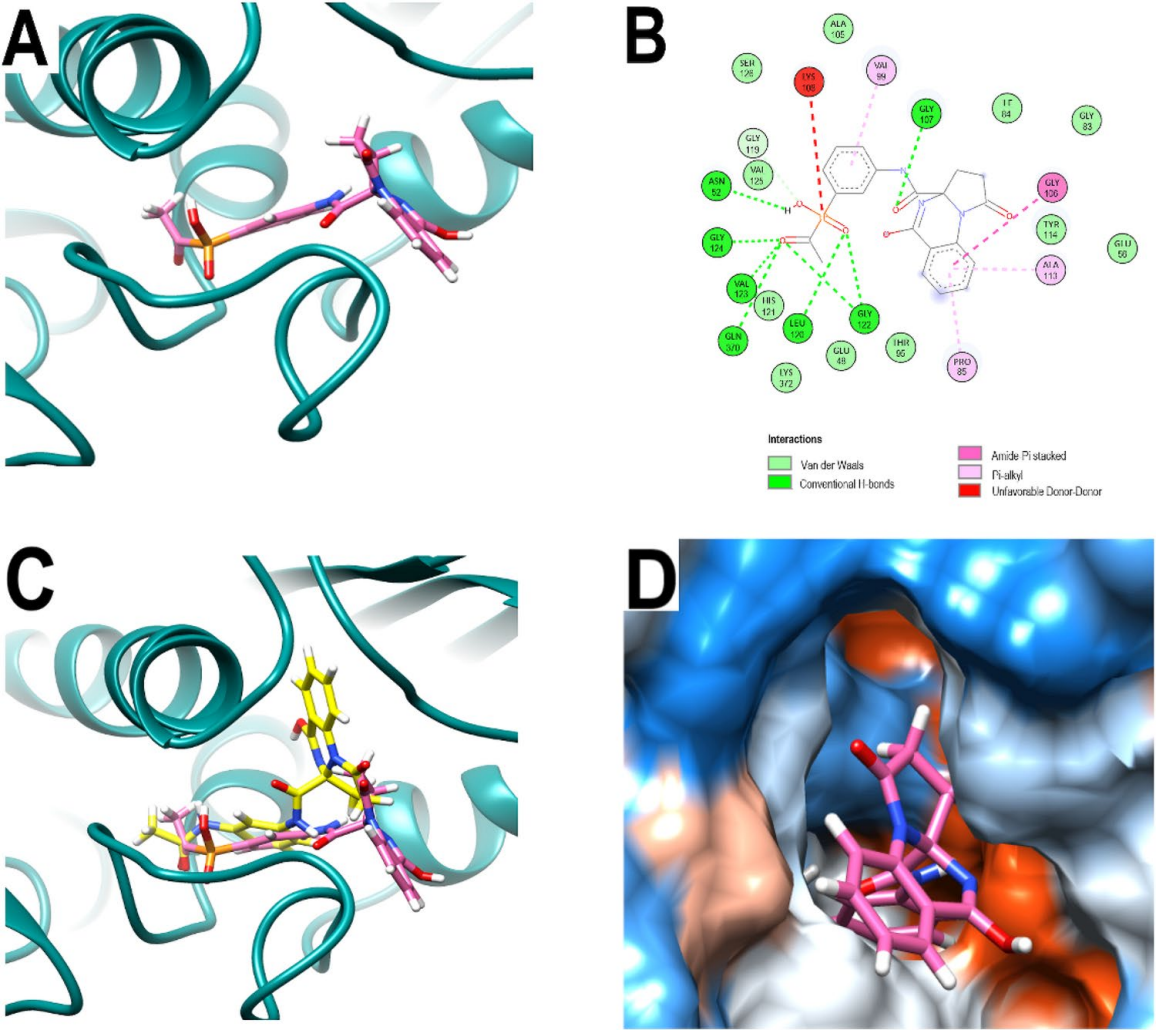
Analysis of the best-predicted docking pose of PQP. (**A**) 3D representation of 3ZKBL-PQP complex. (**B**) 2D interaction diagram of 3ZKBL-PQP complex. (**C**) 3D representation of 3ZKBL-PQP complex superimposed with PQd (yellow backbone). (**D**) Hydrophobic surface representation of 3ZKBL complexed with PQP.

As depicted in Fig. 3A–B, in the catalytic site of 3ZKBL the benzyl of PQP form interactions with VAL99 while its quinazoline backbone interacts with PRO85, ALA113 (π-alkyl), and with GLY106 (amide–π staking). As observed with the hydroxyl of the ribose of ANP, the carbonyl group of the amide linker of PQP forms an H-bond with GLY107. In addition, H-bonds are formed among the carbonyl oxygen of PQP and GLY122, VAL123, and GLY124. Moreover, an additional H-bond appears between this same oxygen and GLN370. Importantly, when analyzing the effect of the incorporated phosphate group, it is possible to observe that it enables the formation of H-bonds involving ASN52, LEU120 and GLY122.

Remarkably, despite the relative similarity in the type of interactions formed by PQd and PQP with 3ZKBL, when comparing the docked poses of the respective pyrrolo[1,2-a]quinazoline backbones, a rotation of approximately 180 degrees is observed (Fig. 3C), with the quinazolinic benzene of PQP partially exposed to the solvent (Fig. 3D).

**Figure 4:**
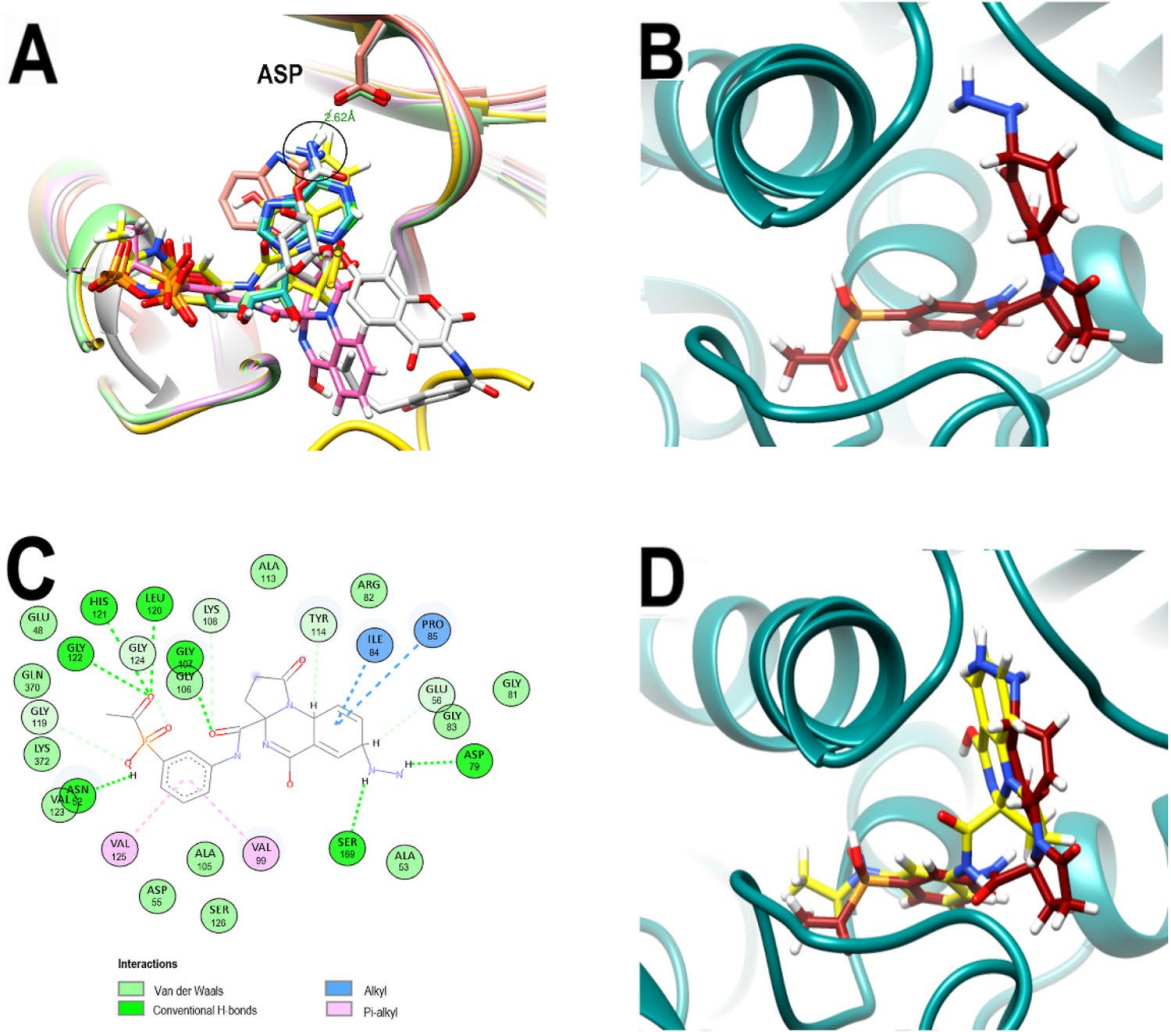
Comparative poses of co-crystallized ligands of diverse GyrBs and analysis of the best-predicted docking pose of PQPNN. (**A**) Computationally performed overlay of PQd and co-crystallized ligand-with GyrBs: ADP (cyan), ATP (light green) AX7 (orange), novobiocin (white), ANP (green), PQd (yellow) and PQP (magenta). The black circle highlights the overlapping nitrogen of the co-crystallized ligands. (**B**) 3D representation of the 3ZKBL-PQPNN complex. (**C**) 2D interaction diagram of the 3ZKBL-PQPNN complex. (**D**) 3D representation of PQPNN-3ZKBL complex superimposed with PQd (yellow backbone).

Concordantly, this backbone pose is also significantly different from that adopted by ANP. Moreover, while backbones of ligands and inhibitors co-crystallized with GyrB of *Escherichia coli* (ADP in PDB: 4PRX), *Staphylococcus aureus* (AX7 in PDB: 5Z9P), *Salmonella enterica* (ATP in PDB: 6J90) and *Mycobacterium smegmatis* (novobiocin in PDB: 6Y8O) overlap when their crystallographic poses are analyzed, the pyrrolo[1,2-a]quinazoline backbone of PQP does not (Fig. 4A). These results together suggested that the new pose of the backbone of PQP might not be the most favorable for its interaction with the enzyme. Furthermore, in all these co-crystallized ligands the presence of an overlapping H-bond donor amino group is identified, forming H-bonds with a conserved ASP residue (ASP79 in 3KZBL and 6Y8O, ASP73 in 4PRX and 6J90, ASP81 in 5Z9P) located at a distance of approximately 2.62 Å.

In order to generate interactions with ASP79 and to resemble in PQP the backbone orientation observed in these ligands, their quinazolinic benzene was modified by adding an amino group to its carbon five. This carbon was selected from the overlap analysis of PQd and co-crystallized ligand poses mentioned above. In addition, to increase the molecular diversity and explore a larger number of possible interactions at the active site of 3ZKBL, the MolOpt server was used to generate a series of analogs from this new amino derivative by bioisosteric substitution of its amino group. Thus, a total of 95 molecules were obtained and subsequently filtered for removal of PAINS and covalent inhibitors to perform virtual screening using LeDock.

Among the derivatives generated, the analog with the best binding energy resulted from the introduction of the –NH–NH2 group on the quinazolinic benzene (Fig. 4B). This ligand (hereinafter called PQPNN) exhibits binding energy of − 11.25 kcal/mol, representing an increase of 2.13 kcal/mol compared to the binding energy of PQd. Notably, the binding energy of PQPNN is even higher than that exhibited by ZINC000012462120, which had the best score in the virtual screening of the initial dataset (− 11.1 kcal/mol, Table S1, LeDock screening).

Moreover, under the conditions of the present study, the affinity of PQPNN is also higher than that of ligands with known pKi values from in vitro studies (best binding energy: ZINC000169340141: − 10.5 kcal/mol). When analyzing its interaction pattern depicted in Fig. 4C, it is possible to observe several H-bonds generated by the carbonyl oxygen (with LEU120, HIS121, and GLY122), the phosphate hydroxyl, and the oxygen in the amide linker. Remarkably, the introduced –NH–NH2 group form an H-bond with ASP79, as the co-crystallized ligands, and also with SER169, allowing the pyrrolo[1,2-a]quinazoline backbone to be oriented like that of the PQd (Fig. 4D).

Notably, the interacting residues ASN52 and ASP79 (*E. coli* numbering: ASN46 and ASP73) are key amino acids to GyrB function and their substitution can cause reduction or abrogation in the activity of the enzyme^28,29^. Therefore, mutations in these residues would not be a successful bacterial mechanism of resistance against PQPNN. In addition, is noteworthy that, among the bonds formed by PQPNN there is no interaction involving ARG141 (*E. coli* numbering ARG136), whose substitution confers bacterial resistance against aminocoumarins antibiotics such as novobiocin^29–31^. Consequently, this resistance mechanism developed by some bacterial strains may not be effective against PQPNN either.

### Pharmacokinetic and toxicological analyses of PQPNN

Considering that PQPNN is an unpublished chemical structure; the next step was to obtain information on some of its pharmacokinetic properties, which are described in Table 2. Predictions performed with the SwissADME server show that, contrary to PQd, PQPNN has low gastrointestinal absorption, so for the next stage of in vivo studies it might be necessary to develop a formulation that allows its parenteral administration. The ability of a drug to cross the blood–brain barrier (BBB) is an indispensable feature of a molecule to provoke effects on the central nervous system^32^. However, when the action of the drug is required in other tissues, its passage through the BBB may cause undesirable effects^32,33^. In the case of PQPNN, it does not present properties that allow it to cross the BBB and generate adverse effects.

**Table 2:** Comparative pharmacokinetics and toxicological analyses of PQd and PQPNN.

	PQd	PQPNN
**Pharmacokinetic analyses**		
GI absorption	**High**	**Low**
BBB permeant	No	No
P-gp substrate	**Yes**	**No**
CYP1A2 inhibitor	No	No
CYP2C19 inhibitor	No	No
CYP2C9 inhibitor	No	No
CYP2D6 inhibitor	No	No
CYP3A4 inhibitor	No	No
**Toxicological analyses**		
Kv11.1 (hERG) inhibition	No	No
Carcinogenicity	No	No
Ames mutagenesis prediction	Negative	Negative

Pharmacokinetics analyses were performed with SwissADME and the toxicological analyses were performed with Pred-hERG and AdmetSAR servers.

The treatment of *M. tuberculosis* infection brings some additional challenges to those faced with other bacterial diseases. Its intracellular nature allows it to efficiently evade the immune system and also to reduce its exposure to effective concentrations of some antibiotics^1^. The efflux pumps are also involved in the reduction of the efficacy of several drugs, including various antituberculosis antibiotics^34^. In addition, some cells infected with *M. tuberculosis* are induced to express efflux pumps such as P-glycoprotein (P-gp)^35^. Notably, SwissADME analyses indicate that, in contrast to PQd, PQPNN is not a substrate of P-gp, a result that reveals an additional advantage of the chemical modifications introduced on the molecule. In addition, PQPNN does not inhibit cytochromes related to drug metabolism, so there would be no likelihood of interactions of PQPNN, through this mechanism, with drugs that are substrates of these enzymes.

One of the important steps in the initial stages of the search and optimization of new drugs is to recognize molecules with the potential to inhibit the voltage-gated potassium channel Kv11.1 encoded by hERG (human ether-a-go-go-related gene). Kv11.1 is involved in cardiac action potential repolarization and its inhibition is related to cardiotoxicity with potentially fatal outcomes^36^. In fact, several previously approved drugs have been withdrawn from the market due to this off-target effect^37^. To predict the ability of PQPNN to inhibit Kv11.1, the Pred-hERG server was used and the results show that the compound is non-cardiotoxic (Fig. S4).

Another crucial aspect that must also be evaluated in a new molecule is its carcinogenicity. The AdmetSAR server was used to analyze this potential risk. The results show that PQPNN is not carcinogenic and also shows negative results for the Ames mutagenesis prediction. These analyses collectively show that PQPNN meets several of the pharmacokinetic and toxicological criteria to continue further in vivo studies. Finally, the synthetic accessibility of this molecule calculated by MolOpt (5.197) and by SwissADME (5.35) on a scale from 1 (very easy) to 10 (very difficult) indicates that PQPNN presents an intermediate complexity for its chemical synthesis.

### Molecular dynamics analyses

**Figure 5:**
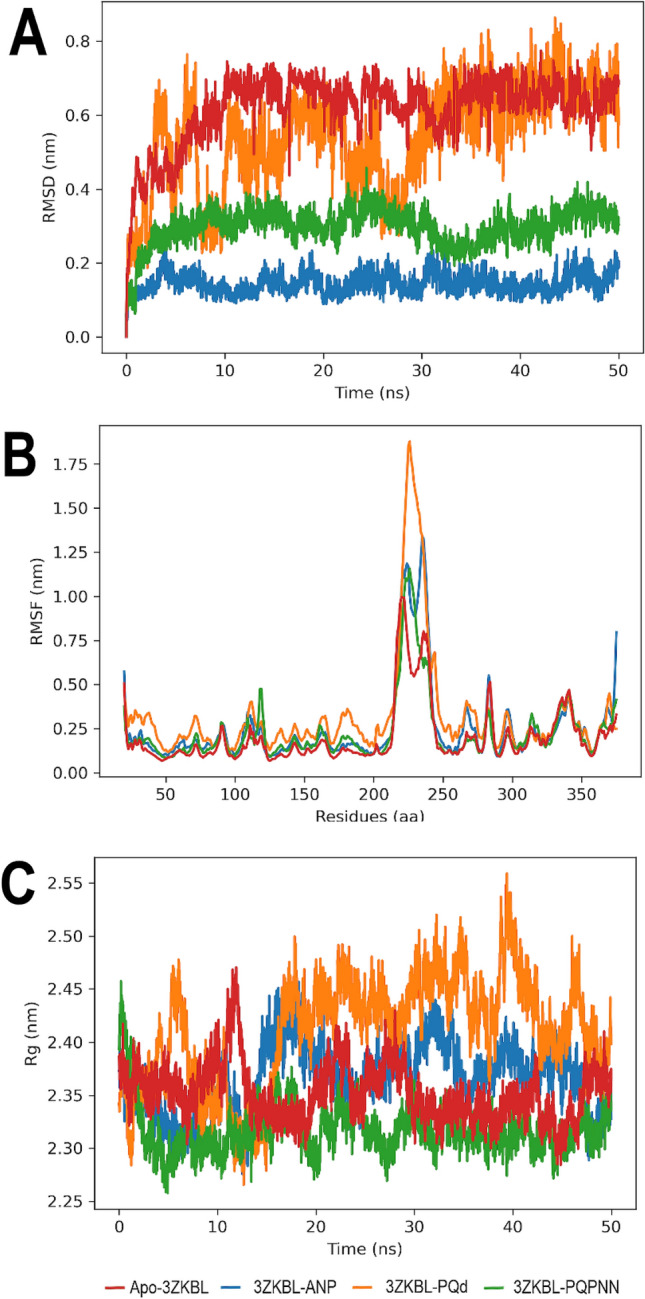
MD analyses of apo-3ZKBL and 3ZKBL complexed to ANP, PQd, and PQPNN. (**A**) RMSD values. (**B**) RMSF values. (**C**) Rg values. All performed with Gromacs.

To further evaluate the potential ability of PQPNN to act as an efficient inhibitor of 3ZKBL, MD simulations were performed. The RMSD value of the apo-3ZKBL shows a progressive increase in the first 10 ns reaching approximately 0.68 nm stabilizing until the end of the run, but fluctuations of around 0.6 to 0.72 nm are observed during most of the simulation. In addition, the MD analysis of the 3ZKBL complexed with ANP was performed to compare it with the dynamic behaviors of the complexes formed with PQd and PQPNN (Fig. 5A). The results of the 3ZKBL-ANP complex show an RMSD value lower than that of the apo-enzyme, exhibiting an average of approximately 0.15 nm during the entire run, revealing that the presence of the co-crystallized ligand stabilizes the structure of the enzyme. On the other hand, the complex 3ZKBL-PQd reaches RMSD values between 0.4 and 0.68 during the first 30 ns, but with stronger fluctuations throughout the simulation. In fact, at only two ns the complex reaches an RMSD of 0.6 nm but then decreased to values of about 0.37 nm (4.8–5.2 ns), 0.25 nm (7–10 ns), and 0.38 nm (26–28 ns) until reaching again an RMSD value of about 0.68 nm in the last 20 ns.

These results show that, compared to ANP, PQd forms a complex with lower stability. To further study the behavior of the 3ZKBL-PQd complex, the orientation adopted by PQd was analyzed in representative snapshots of these fluctuations during the run (5 and 45 ns). As shown by comparing the ligand pose at 5 ns (Fig. S5A) and 45 ns (Fig. S5B) it fluctuates in the orientation of the pyrrolo[1,2-a]quinazoline backbone indicating that its interactions with the enzyme are not sufficient to limit its free rotation.

On the other hand, the 3ZKBL-PQPNN complex reveals an evident decrease in RMSD values compared to that of the complex with PQd. In the first 5 ns, the complex slowly increases the RMSD value until 0.31 nm and stabilizes until practically the end of the simulation, with a slight decrease to 0.23 nm between 32 and 36 ns. The snapshots obtained at 5 ns (Fig. S5C) and 45 ns (Fig. S5D) show that the pyrrolo[1,2-a]quinazoline backbone retains its orientation in the active site of 3ZKBL, which must contribute to the higher stability of this complex. These results are in good agreement with those observed in the docking analyses and confirm the improved structural features of PQPNN.

The analysis of the RMSF obtained from the apoenzyme, as well as from its complexes with ANP, PQd, and PQPNN show values from approximately 1.0 to 1.7 nm among the amino acids 210 to 248, a region that corresponds to a loop and whose high flexibility may explain these behaviors^38^. In addition, in the case of PQd, it is observed that its interactions with 3ZKBL generate increased RMSF values indicating a greater fluctuation of amino acid residues, especially those close to the active site of the enzyme, which could be related to the mobility of the ligand in this region as mentioned above. On the other hand, overall, it can be observed that both ANP and PQPNN have an RMSF profile quite similar to that of the apo-enzyme (Fig. 5B).

The next step was to analyze the Rg values of apo-3ZKBL and its complexes to examine their changes in compactness throughout the simulation. Fig. 5C shows that the non-complexed enzyme exhibits an average Rg value of approximately 2.35 nm over the 50 ns, but values of 2.4 nm (10, 22, 28, and 48 ns) and up to 2.44 nm (11 ns) are observed. The Rg values of the complex formed with PQd show wide fluctuations throughout the run presenting values between 2.29 nm (0.6, 12, 12.5, and 14.9 ns) and 2.55 nm (39 ns) with an average Rg of about 2.43 nm. The complex formed between the enzyme and ANP presents a mean value of 2.37 nm throughout the analysis, with a narrower fluctuation range compared to the complex with PQd. The upper and lower limits are 2.27 and 2.45 nm at 12.5 and 16.25 ns, respectively. Notably, when analyzing the Rg of the complex formed with PQPNN, there is evidence of greater stability, revealing a mean value of approximately 2.32 nm with slight fluctuations over 50 ns. Moreover, this complex shows even greater compactness than that exhibited by the apoenzyme. All these results demonstrate that the complex formed by PQPNN and 3ZKBL shows high stability.

### Hydrogen bonds analyses

**Figure 6:**
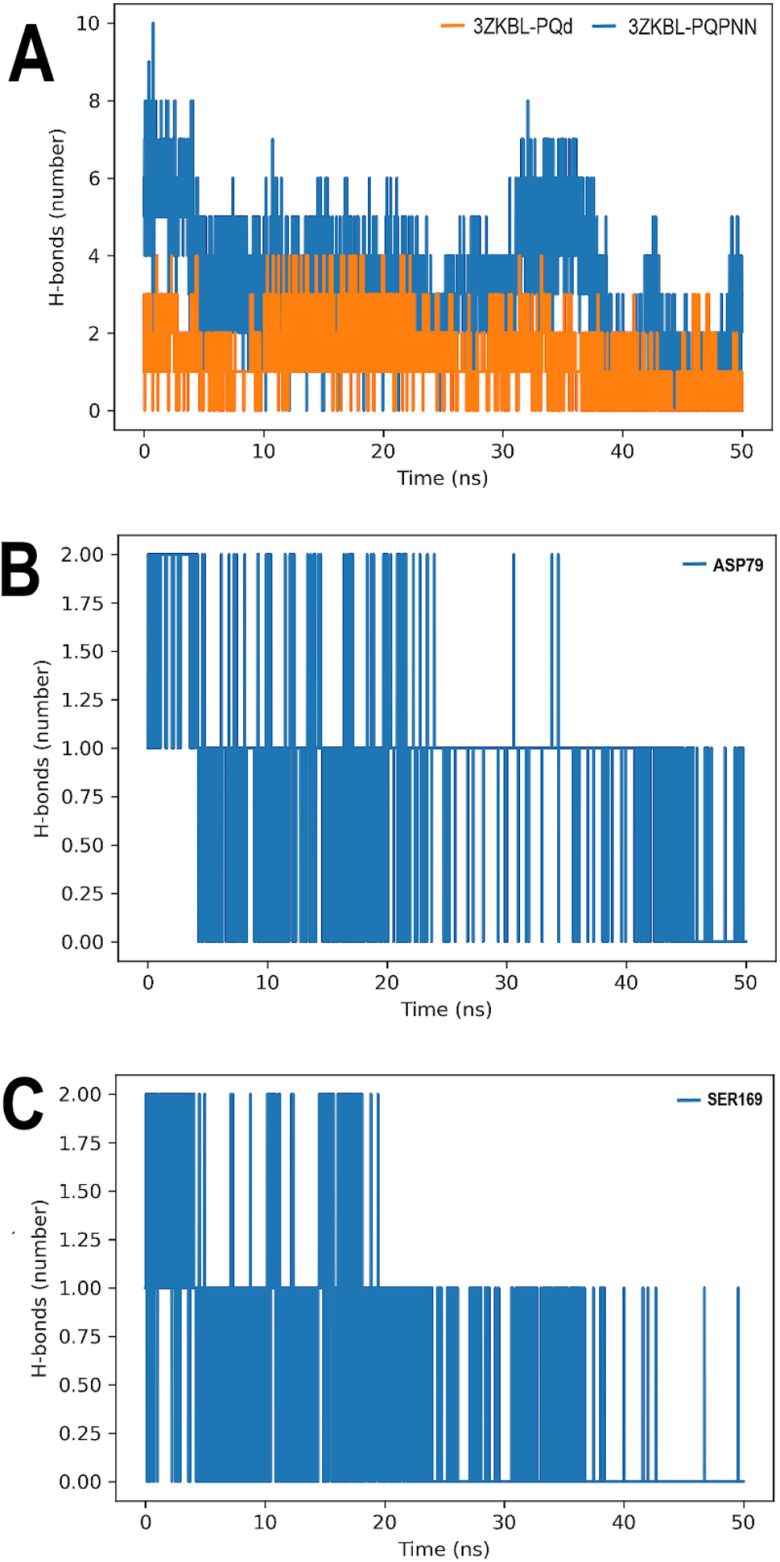
H-bonds analyses of 3ZKBL complexed with PQd, and PQPNN. (**A**) H-bonds in complexes. (**B**) Involvement of ASP79 of the 3ZKBL-PQPNN complex in H-bond formation during MD simulation. (**C**) Involvement of SER169 of the 3ZKBL-PQPNN complex in H-bond formation during MD simulation. All performed with Gromacs.

The formation of H-bonds was also examined from MD simulations to expand the understanding of previous findings. As shown in Fig. 6A, PQd establish two and three H-bonds with 3ZKBL during the first 36.5 ns, occasionally reaching up to four interactions, thereafter forming mainly one and two bonds until the end of the run. Analysis of PQPNN interactions shows that during the first 4.5 ns an average of six H-bonds are formed, reaching a maximum of up to 10 interactions. From this time until 31 ns mainly three and four H-bonds are formed, followed by an interval of approximately seven ns in which five to six H-bonds are established. However, between 23 and 25.5 ns is more frequent the formation of two H-bonds. Finally, from 38 ns until the end of the simulation, two H-bonds on average are formed. Remarkably, when the involvement of individual amino acids is analyzed, it is possible to verify that both ASP79 and SER169 actively participate in the formation of H-bonds during most of the simulation, confirming the molecular docking results of the PQPNN-3ZKBL complex (Fig. 6B,C).

With the resurgence of *M. tuberculosis* on the global public health scene, with strains increasingly resistant to traditional antibiotics, in silico approaches represent an advantageous option with less cost and time to select the most promising inhibitors of bacterial enzymes. In this context, the present study screened for potential *Mt*GyrB inhibitors from 5462 selected natural products. The consensus scores between LeDock and Vina show PQd as the most promising ligand in the dataset. However, its subsequent chemical optimization by replacing an NH with a phosphate group and incorporating a -NH-NH2 group resulted in the PQPNN derivative, which exhibits higher affinity and superior stability with 3KZBL than the parent compound, as demonstrated by molecular docking and MD analyses.

Furthermore, PQPNN interacts with several amino acids critical for the *Mt*GyrB ATPase activity, without involving bonds with ARG141, a substitution that is a frequent cause of bacterial resistance to aminocoumarin antibiotics. Finally, the ADME and toxicological analyses show that this molecule meets several of the criteria to be considered suitable for further studies in vivo. Given the encouraging results presented here, we consider that the next step should be to evaluate in vitro the ability of PQPNN to inhibit *Mt*GyrB and test their potential antibacterial activity.

## Methods

### Target and ligands preparation

For the present work, the 3D X-ray diffraction structure of *Mt*GyrB (PDB ID: 3ZKB (resolution: 2.9 Å, free R-value: 0.240, working R-value: 0.182, observed R-value: 0.184), chain D), and it co-crystallized ligand, ANP^39^, were downloaded from RCSB Protein Data Bank in July 2021. The low electron density of the 3ZKB crystal between 216–239 amino acid sequences was rebuilt by fragment assembly simulation using the Modeller-9.22 module implemented in UCSF Chimera tool-1.15 version^40^. In total 500 models were generated with DOPE-HR as a loop modeling protocol with zDOPE score and RMSD estimation. In addition, the protein model with the rebuilt loop was stereochemical validated by generating a Ramachandran diagram on the MolProbity server and also with ERRAT^41^, PROVE^42^, and VERIFY^43^ scores from the Structure Analyses and Verification Server-6 version (SAVES), to analyze non-bonded interactions, calculate atom volume and also atomic compatibility between 1D amino acid sequence and 3D model of 3ZKBL. Subsequently, the Dock Prep module of UCSF Chimera was used as default.

An initial 20,098 natural product structures were selected from the Alinda Biogenic catalog in the UCSF ZINC 15 database in July 2021. Subsequently, they were filtered using the FAF-Drugs4 server^44^ to remove PAINS, covalent inhibitors and to select only molecules that meet Lipinski rule of five^45^ resulting in 5,462 molecules for MDVS. Were used as controls the co-crystallized ligand, ANP, (ZINC000008660410 ([[[[(2R,3S,4R,5R)-5-(6-aminopurin-9-yl)-3,4-dihydroxyoxolan-2-yl]methoxy-hydroxyphosphoryl]oxy-hydroxyphosphoryl]amino]phosphonic acid) and the physiological ligand ATP (ZINC000011524400 (1-adenin-9-yl-1-deoxy-5-O-triphospho-beta-D-ribo-pentofuranose)), were also used as controls two inactive compounds tested in vitro by the ATPase assay of GyrB from *E. coli*^38^: ZINC000001688375 (2-chloro-5-nitroaniline) and ZINC000003861263 (4-amino-1H-imidazole-5-carboxamide)). All ligands were downloaded in SDF format, then the hydrogens were assigned to the structures at pH 7.4 and 1,000 steps of minimization of these structures were performed with the MMFF94 force field using the conjugate gradient algorithm and transformed into PDBQT and MOL2 formats using the Open Babel-3.1.1 tool version.

### Molecular docking-based virtual screening analyses

The MDVS analyses were performed using three freely software available to the academic users: Auto-Dock Vina-1.1.2 version^46^, LeDock-1.0 version^47^, and PLANTS-1.2 version^48^. For Vina analysis, 3ZKBL was converted in PDBQT file format using the Auto-DockTools-1.5.6 version^49^, the runs were set with exhaustiveness of eight, the grid box dimensions were set to 30 × 30 × 30 Å, the grid coordinates of the active site (x, y, and z axes, respectively) were set to -26.86, -27.11, and 17.77. For LeDock, 3ZKBL was previously processed by the LePro tool. The binding pocket was set to -41.86 and -11.86 for x, -42.11, and -12.11 for y and 2.77 and 32.77 for z coordinates. All other parameters were set to default for sampling by a combination of simulated annealing and evolutionary optimization. For PLANTS, 3ZKBL was processed by the SPORES 1.3 tool centered on the same coordinates described for Vina and with a radius of 20 Å. The search speed was set to one and the scoring function was selected as ChemPLP. For all software the clustering RMSD was set to 2.0 Å and all docking scores were calculated by the default scoring function.

Before carrying out virtual screening with the selected ligands, it was validated the ability of the three software to reproduce the crystallographic pose of the ligand co-crystallized with 3ZKB, ANP. The DockRMSD server^50^ was used to calculate the RMSD values. To define the most promising ligand from the three virtual screenings, a rank-by-rank selection criterion was used among the scores generated by the software with the best Pearson correlation, and a *p*-value with *p* < 0.05 was considered significant.

The binding energy analyses, visualizations and, correlations analyses were done using Python custom scripts in conjunction with Pandas, NumPy, Matplotlib, and Seaborn libraries. The 2D analyses of the interaction patterns in protein–ligand complexes was performed and visualized using Discovery Studio Visualizer-2021 version. In all these analyses, the default cutoff interaction distances were used, except for H-bond visualization, which was set to a maximum distance of 0.35 nm (default cutoff distance for MD molecular dynamics analyses in Gromacs software). The 3D visualizations and analyses were performed with UCSF Chimera and the chemical structure of ligands was done with the Marvin JS tool.

### Evaluation of software performances from virtual screenings using ligands with known pKi values

To perform a further step of evaluation of the software used in the present work was conducted virtual screenings of molecules with known pKi values from in vitro inhibition assays against *Mt*GyrB ATPase. For this purpose, 140 molecules were retrieved from the UCSF ZINC 15 database and subsequently prepared as described in the topic Target and ligands preparation. Finally, virtual screenings were conducted with LeDock and Vina as described in the topic Molecular docking-based virtual screening analyses.

### Structure optimization

Ligand optimization was assisted by the MolOpt server for bioisosteric replacement^51^ using the simplified molecular-input line-entry system (SMILES) notation of the amino derivative of the PQP ligand.

### Pharmacokinetic and toxicological predictions

To obtain information on the physicochemical and pharmacokinetic properties of the most promising molecules in this study, the Swiss-ADME server was used^52^. Predictions of the ability of the ligands to inhibit Kv11.1 were performed using the Pred-hERG server^53^. The carcinogenic and mutagenic risk was analyzed by using the AdmetSAR server^54^. For all these analyses the respective SMILES codes were used: PQd CC(= O)Nc1cccc(NC(= O)[C@]23CCC(= O)N2c2ccccc2C(= N3)O)c1 and PQPNN: CC(= O)[P@@](= O)(O)c1cccc(NC(= O)[C@]23CCC(= O)N2[C@H]2C = C[C@@H](NN)C = C2C(= N3)O)c1.

### Molecular dynamic simulations

The best docking poses calculated with LeDock (with the lowest binding energy) of PQd, of its optimized derivatives, PQPNN, and also of the co-crystallized ligand, ANP, complexed with 3ZKBL were selected to perform the MD analyses. In addition, MD analyses were performed with apo-3ZKBL. All these simulations were executed with Gromacs 2021.1^55^. Simulation conditions were all-atom CHARMM 36 force field^56^, transferable intermolecular potential water model 3P (TIP3P), and all simulations maintained neutral ionization with Na + or Clˉ added to balance the systems, which were all performed in a triclinic box. A total of 50,000 minimization steps were performed using the steepest descent algorithm and long-range electrostatic force using the Particle Mesh Ewald (PME) method. After minimization, two consecutive equilibration episodes and one production episode were performed, all using the Leap-frog algorithm and Berendsen coupling to control pressure and temperature. The first equilibrium simulation was run for 250 ps at 310 K in NVT, followed by 1 ns in NPT at 1.0 bar sets. The production simulations were 50 ns long and coordinates were saved every 10 ps. The LINCS algorithm implementation was used to regulate the covalent bonds. All trajectories were corrected for the edge effect of the periodic conditions.

The analyses performed were RMSD, RMSF, and Rg, all performed considering the protein backbone alone or with the ligand, and H-bonds considering protein and ligand, all obtained from Gromacs scripts in conjunction with the NumPy, Pandas, Matplotlib, Seaborn, Pytraj libraries, and the Xmgrace tool.

## Supplementary Information

Supplementary Information 1.

Supplementary Information 2.
