# Supplementary material for: Virtual screening, optimization and molecular dynamics analyses highlighting a pyrrolo[1,2-a]quinazoline derivative as a potential inhibitor of DNA gyrase B of Mycobacterium tuberculosis
Source: Sci Rep. 2022 Mar 18;12:4742. doi: 10.1038/s41598-022-08359-x (PMC8933452; doi:10.1038/s41598-022-08359-x)
Supplement: Supplementary file 1 — Supplementary Information 1. [file 41598_2022_8359_MOESM1_ESM.pdf]

Supplementary information

Virtual screening, optimization and molecular dynamics analyses highlighting a pyrrolo[1,2-a]quinazoline derivative as a potential inhibitor of DNA gyrase B of *Mycobacterium tuberculosis*

Juan Marcelo Carpio Arévalo[1], *Juliana Carolina Amorim*[1,*]

[1] Academic Unit of Health and Wellness, Catholic University of Cuenca, Cuenca, Azuay, Ecuador

[*] Corresponding author address: julianacarolinaamorim@gmail.com

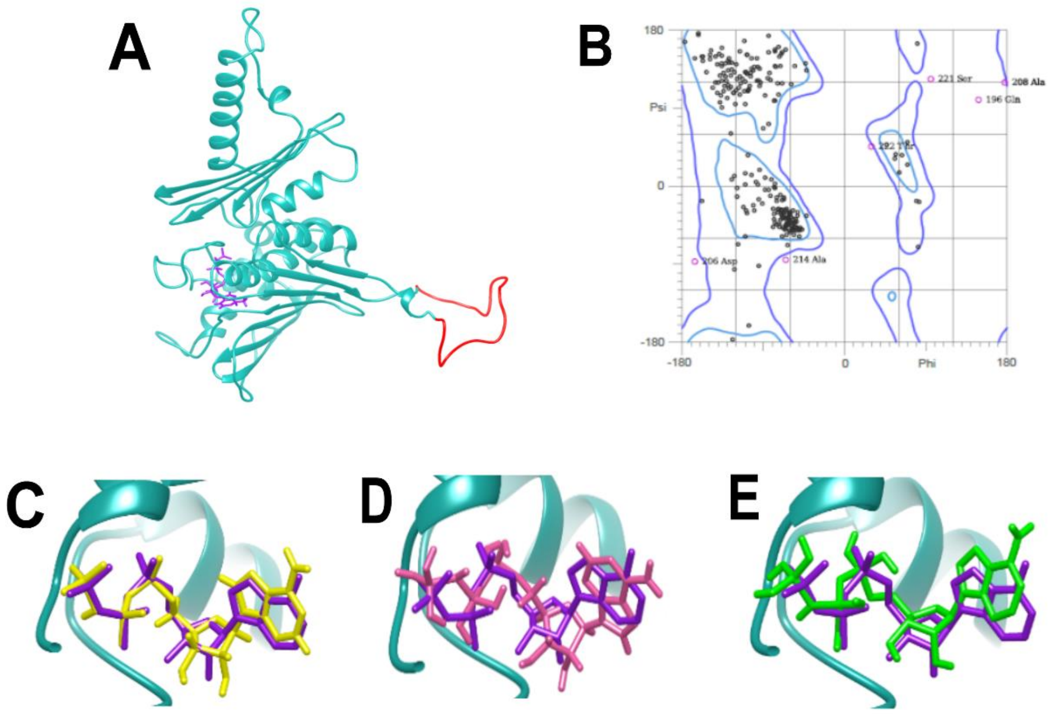

Fig. S1. Model of 3ZKB and redocking of the co-crystallized ligand using LeDock, PLANTS, and Vina. A) 3D structure of 3ZKB co-crystallized with ANP (highlighted with magenta color) with loop rebuilt region of residues 216–239 (highlighted with red color). Models were rebuilt using the Modeller tool implemented on UCSF Chimera. B) Ramachandran plot for 3ZKBL using MolProbity tool. C) ANP redocking with 3ZKBL using LeDock software. D) Using PLANTS software. E) and also using Vina software.

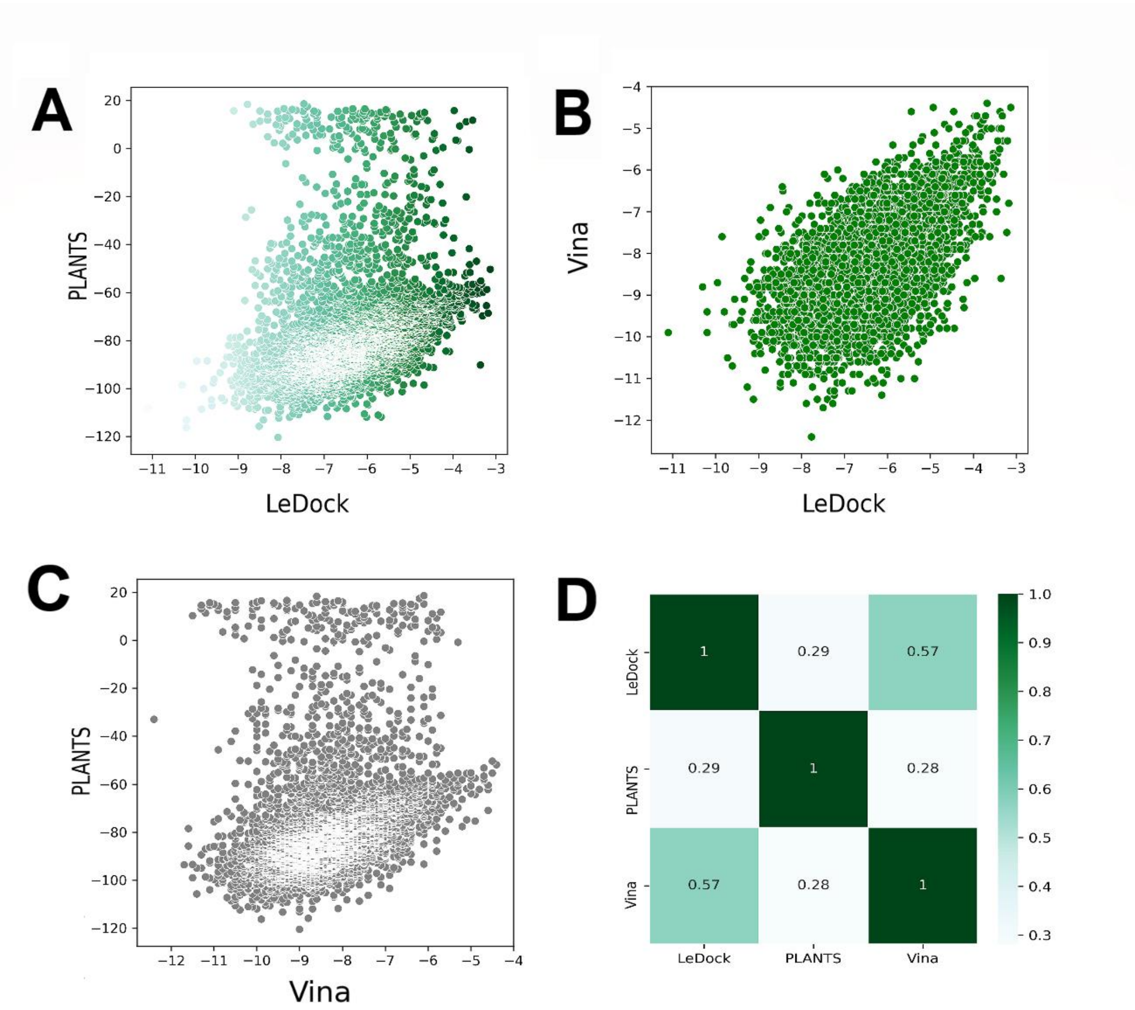

Fig. S2. Correlations among ligands using LeDock, PLANTS, and Vina. A) Scatter plot representing the correlations between the scores of 5,462 ligands and four controls analyzed with LeDock and PLANTS. B) Using LeDock and Vina. C) And also using Vina and PLANTS. D) Pearson correlation between the scores generated by the software used in this study

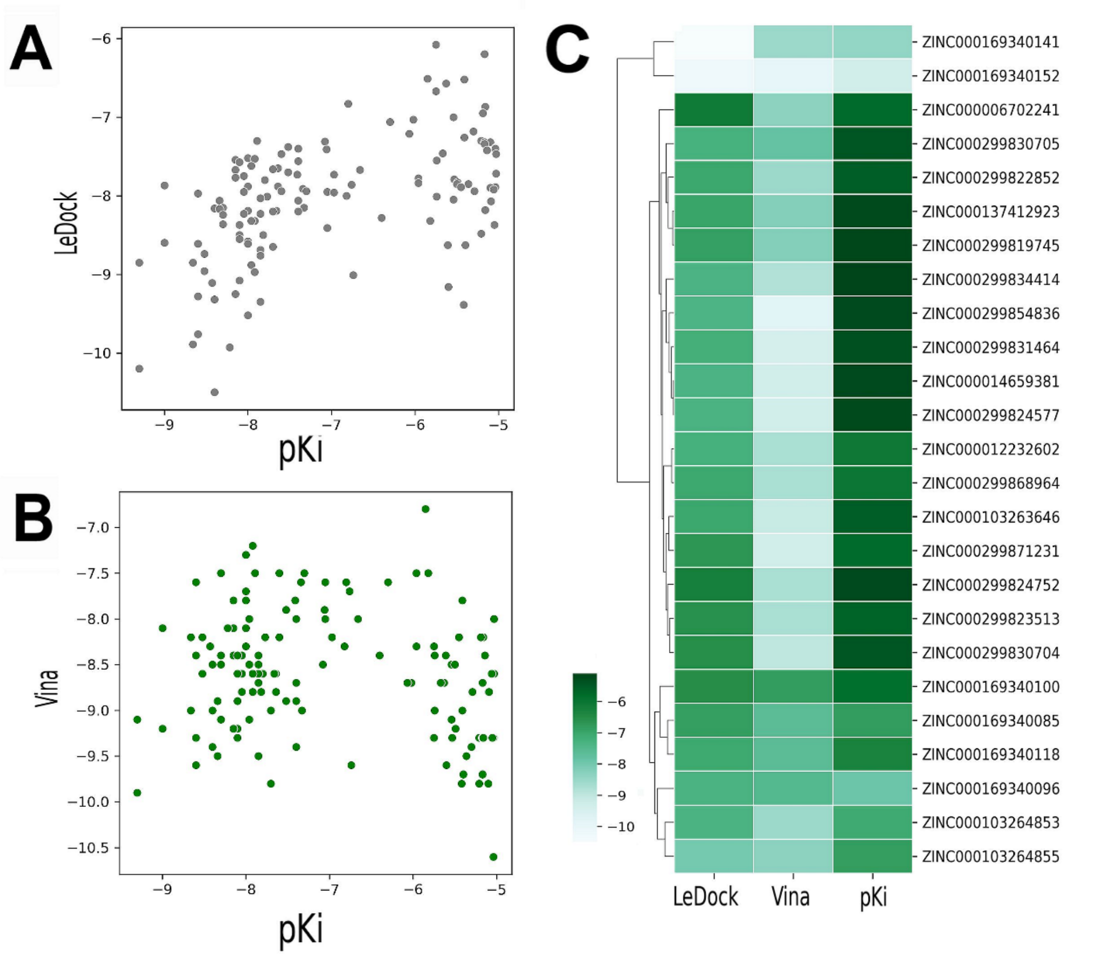

Fig. S3. Virtual screening of ligands with known pKi values obtained in *Mt*GyrB inhibition assays. A) Correlation between in vitro and in silico results of experimentally tested ligands using LeDock. B) And using Vina. C) Heatmap of the 25 best-scored results of experimentally tested ligands using LeDock, Vina, and pKi values.

RESEARCH OUR TEAM WEB APPS HOT PUBLICATIONS GET IN TOUCH NEWS

Pred-hERG Results

Prediction / Potency	Confidence	Applicability domain (AD)	Probabilty Map
Non-cardiotoxic (-)	60%	No<br>Value=0.25 and limit= 0.26	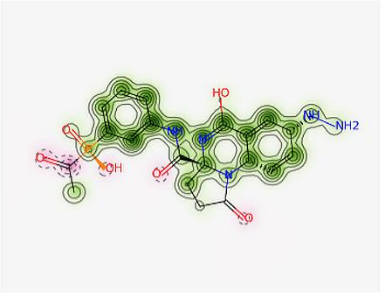
Not applicable	Not applicable		

Fig. S4. Prediction of the capacity of PQPNN to inhibit Kv11.1 (hERG) using Pred-hERG server

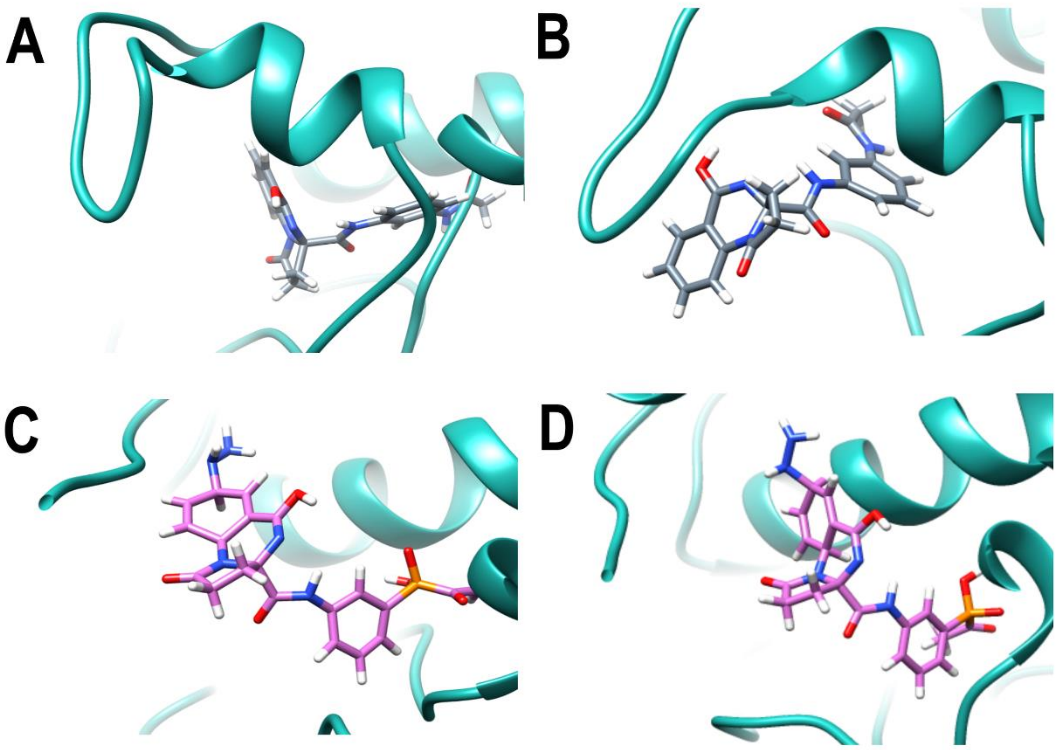

Fig. S5. Representative MD snapshots of 3ZKBL complexed with PQd and PQPNN. A) 3ZKBL-PQd snapshot at 5 ns. B) 3ZKBL-PQd snapshot at 45 ns. C) 3ZKBL-PQPNN snapshot at 5 ns. D) 3ZKBL-PQPNN snapshot at 45 ns.
